# Construction and Validation of a TyG-ALT-Based Diagnostic Risk-Stratification Model for Metabolic-Associated Fatty Liver Disease in Patients with Obstructive Sleep Apnea

**DOI:** 10.3390/jcm15145346

**Published:** 2026-07-08

**Authors:** Xiaohui Wang, Lihua Deng, Ya’nan Wei, Qian Xue, Meiqi Liu, Jianping Zhang, Jingtong Wang

**Affiliations:** 1 Department of Geriatrics, Peking University People’s Hospital, Beijing 100044, China; 2 Shijiazhuang People’s Hospital, Shijiazhuang 050011, China

**Keywords:** obstructive sleep apnea, metabolic-associated fatty liver disease, triglyceride-glucose index, alanine aminotransferase, diagnostic risk-stratification model

## Abstract

**Objective:** The objective of this study is to investigate the clinical value of the triglyceride-glucose (TyG) index combined with alanine aminotransferase (ALT) in identifying prevalent metabolic-associated fatty liver disease (MAFLD) in patients with obstructive sleep apnea (OSA), and to construct and validate a diagnostic risk-stratification model. **Methods:** Clinical data of OSA patients were retrospectively collected from two centers: the Department of Geriatrics, Peking University People’s Hospital (August 2021 to December 2025) and the Department of Geriatrics, Shijiazhuang People’s Hospital (June 2023 to December 2025). MAFLD was diagnosed by abdominal ultrasonography performed by experienced radiologists blinded to laboratory results. Candidate predictors were selected using univariate logistic regression, LASSO regression, and bootstrap stability testing. Model performance was assessed by discrimination (area under the receiver operating characteristic curve, AUC), calibration (Hosmer–Lemeshow test, calibration curves), and clinical utility (decision curve analysis). Internal validation was performed using 10-fold cross-validation and bootstrap resampling with optimism correction. External validation was conducted in an independent cohort. Sensitivity analyses included subgroup analyses stratified by sex, age, BMI, and OSA severity. **Results:** A total of 962 patients were included in the development set and 116 in the external validation set. Multivariate analysis identified TyG index (OR = 1.95, 95% CI: 1.51–2.53), LDL-C (OR = 1.24, 95% CI: 1.02–1.49), BMI (OR = 1.21, 95% CI: 1.16–1.26), and ALT (OR = 1.04, 95% CI: 1.02–1.05) as variables independently associated with prevalent MAFLD, while platelet-to-lymphocyte ratio (PLR) was protective (OR = 0.996, 95% CI: 0.993–0.999). The simplified TyG-ALT model achieved an AUC of 0.714 (95% CI: 0.685–0.744) in the development set, with an optimism-corrected AUC of 0.712, and an AUC of 0.783 (95% CI: 0.701–0.866) in external validation. The model demonstrated good calibration and favorable clinical net benefit within the threshold range of 0.30–0.70. The optimal cutoff was 0.564, with sensitivity of 68.7% and specificity of 79.6%. **Conclusions:** The TyG-ALT model demonstrates good discriminative ability, calibration, and clinical utility for case-finding and risk stratification of prevalent MAFLD in OSA patients, particularly for identifying high-risk individuals requiring confirmatory imaging.

## 1. Introduction

MAFLD, previously known as non-alcoholic fatty liver disease, is the most common cause of chronic liver disease [1,2,3], affecting more than one-third of the global population [4]. Its disease spectrum includes metabolic-associated simple steatosis, metabolic-associated steatohepatitis, liver fibrosis, cirrhosis, and even hepatocellular carcinoma [5]. Although the progression of fatty liver disease is relatively slow, multiple studies have confirmed that MAFLD accelerates the progression of liver fibrosis and is closely associated with the development of cirrhosis and hepatocellular carcinoma [6,7,8,9]. Furthermore, studies have shown that MAFLD is also associated with an increased risk of extrahepatic events, such as atherosclerotic cardiovascular disease, reflux esophagitis, chronic obstructive pulmonary disease, esophageal cancer, and gastric cancer [10,11,12,13].

Obstructive sleep apnea refers to apnea and hypopnea caused by upper airway collapse during sleep, and is a risk factor for various clinical conditions in adults, including hypertension, diabetes, and cardiovascular and cerebrovascular diseases [14,15]. The global prevalence of OSA is approximately 936 million, with China having the highest number of cases at 176 million [16]. With the increasing prevalence of overweight and obesity, the incidence of OSA is rising annually, making it a significant public health issue.

A growing body of research has confirmed an association between MAFLD and obstructive sleep apnea–hypopnea syndrome (OSAHS) [17,18,19,20]. However, MAFLD typically presents with symptoms only at advanced stages. Conventional diagnostic methods such as liver biopsy, ultrasound, computed tomography (CT), or magnetic resonance imaging (MRI) often incur high medical costs or carry procedural risks. Therefore, exploring accurate non-invasive diagnostic tools for case-finding of prevalent fatty liver, particularly in high-risk populations such as those with OSAHS, is of paramount importance [21].

In recent years, the triglyceride-glucose (TyG) index has gained increasing attention in the context of disorders related to glucose and lipid metabolism. Studies have shown that the TyG index can be used to assess insulin resistance and identify the risk of various diseases, including MAFLD and cardiovascular disease [22,23,24]. Moreover, combined indices such as TyG index combined with neck circumference and Epworth Sleepiness Scale (ESS) score, or TyG combined with obesity indices like TyG-BMI and TyG-WC have demonstrated superior diagnostic performance compared to the TyG index alone [25,26,27]. Recent studies have emphasized the role of integrated metabolic-inflammation indices for non-invasive risk stratification in metabolic liver disease [28], supporting the rationale for combining metabolic biomarkers such as TyG with hepatic injury markers such as ALT. Emerging evidence also supports the utility of biomarker-based panels for MAFLD screening in high-risk populations, particularly when imaging resources are limited [29]. This study conducts a multicenter investigation into the case-finding of prevalent MAFLD in patients with OSA using a simple diagnostic model based on alanine aminotransferase, triglycerides, and fasting plasma glucose, aiming to facilitate timely diagnosis and intervention, thereby reducing disease burden.

## 2. Subjects and Methods

### 2.1. Study Subjects and Inclusion/Exclusion Criteria

**Study subjects:** This study consecutively enrolled hospitalized patients who underwent polysomnography (PSG) or portable monitoring (PM) at the Department of Geriatrics, Peking University People’s Hospital (August 2021 to December 2025) and the Department of Geriatrics, Shijiazhuang People’s Hospital (June 2023 to December 2025).

**Inclusion criteria:** 1. Diagnosis of OSA defined as an apnea–hypopnea index (AHI) ≥ 5 events/h according to the Guideline for Primary Care of Adult Obstructive Sleep Apnea (2018). 2. Hospitalized patients with complete laboratory and examination results, including liver and kidney function tests, lipid profile, abdominal ultrasound, and others. 3. All included patients underwent abdominal ultrasonography for MAFLD diagnosis; patients diagnosed solely by histopathology or with mixed diagnostic approaches were excluded to maintain a uniform reference standard.

**Exclusion criteria:** 1. Excessive alcohol consumption (weekly ethanol intake ≥ 210 g in men and ≥140 g in women). 2. Presence of other causes of fatty liver, including genotype 3 HCV infection, drug-induced fatty liver, Wilson’s disease, and malnutrition. 3. Incomplete clinical data.

### 2.2. Data Collection

Data collected included: General information (sex, age, height, weight, fatty liver, hypertension, antihypertensive medication, diabetes, hypoglycemic medication, coronary heart disease, etc.); sleep monitoring results (REI, OAI, CAI, ODI, lowest oxygen saturation, mean oxygen saturation, longest apnea time, longest hypopnea time); and laboratory findings (ALT, AST, GGT, Alb, TBIL, Cr, eGFR, UA, FPG, TC, TG, HDL-C, LDL-C, BNP, WBC, LY, Hb, PLT, MPV, CRP).

### 2.3. Disease Definitions

OSA was diagnosed according to the Guideline for Primary Care of Adult Obstructive Sleep Apnea (2018): (1) Presence of any of the following symptoms: (a) Daytime sleepiness, unrefreshing sleep, fatigue, or insomnia; (b) waking due to gasping, choking, or suffocation during sleep; (c) habitual snoring or witnessed apnea; and (d) hypertension, coronary heart disease, stroke, heart failure, atrial fibrillation, type 2 diabetes mellitus, mood disorders, or cognitive impairment. (2) PSG or PM monitoring: AHI ≥ 5 events/h, predominantly obstructive events. (3) In the absence of the above symptoms, PSG or PM monitoring: AHI ≥ 15 events/h, predominantly obstructive events. Diagnosis of adult OSA can be made if criteria (1) and (2) are met, or if criterion (3) alone is met.

MAFLD was diagnosed according to the *Guideline for the Prevention and Treatment of Metabolic-Associated (Non-Alcoholic) Fatty Liver Disease (2024 Edition)*: Abdominal ultrasonography (USG) was used as the imaging modality for diagnosing hepatic steatosis. USG was performed by experienced radiologists, and the diagnosis was based on characteristic sonographic features including increased hepatic echogenicity compared with the renal cortex, attenuation of the ultrasound beam, and poor visualization of intrahepatic vessel borders and diaphragm. Histopathological evidence (liver biopsy) was not used as a routine diagnostic method in this retrospective cohort; patients with biopsy-based diagnosis alone were excluded to ensure reference standard uniformity. In addition to imaging evidence of steatosis, the diagnosis required the presence of at least one component of metabolic syndrome: (1) Overweight/obesity: BMI ≥ 24.0 kg/m^2^, or waist circumference ≥ 90 cm in men and ≥85 cm in women, or elevated body fat content/percentage. (2) Elevated arterial blood pressure/hypertension: arterial blood pressure ≥ 130/85 mmHg, or undergoing antihypertensive treatment. (3) Prediabetes or type 2 diabetes: fasting plasma glucose ≥ 6.1 mmol/L, or 2 h post-load glucose ≥ 7.8 mmol/L, or HbA1c ≥ 5.7%, or history of type 2 diabetes, or HOMA-IR ≥ 2.5. (4) Elevated serum triglycerides: fasting serum TG ≥ 1.70 mmol/L, or undergoing lipid-lowering treatment. (5) Reduced serum high-density lipoprotein cholesterol: serum HDL ≤ 1.0 mmol/L in men and ≤1.3 mmol/L in women, or undergoing lipid-lowering treatment.

Ultrasound has moderate sensitivity (approximately 60–90%) for detecting mild steatosis (<20% liver fat content) and cannot reliably assess steatohepatitis or fibrosis. Magnetic resonance imaging proton density fat fraction (MRI-PDFF) and controlled attenuation parameter (CAP) from vibration-controlled transient elastography were unavailable in our routine clinical setting. This represents an important limitation that may affect the detection of mild steatosis cases. Formal interobserver reliability assessment (e.g., kappa statistics) was not performed in this retrospective cohort. However, all examinations were interpreted by experienced radiologists following institutional standardized reporting templates.

Overweight in adults was defined as BMI ≥ 24 kg/m^2^ according to the National Technical Guideline for Comprehensive Management of Obesity in Primary Care (2025). Participants were categorized into BMI < 24 kg/m^2^ and BMI ≥ 24 kg/m^2^ groups.

### 2.4. Study Design and Statistical Analysis

This was a retrospective cross-sectional study. The model estimates the probability of prevalent MAFLD at the time of clinical assessment rather than predicting future incident MAFLD. For clarity, this manuscript uses “metabolic-associated fatty liver disease (MAFLD)” per the 2024 Chinese guideline terminology and “obstructive sleep apnea (OSA)” per international consensus. The term “OSAHS” appears only in direct quotes from guidelines or historical references.

**Figure 1:**
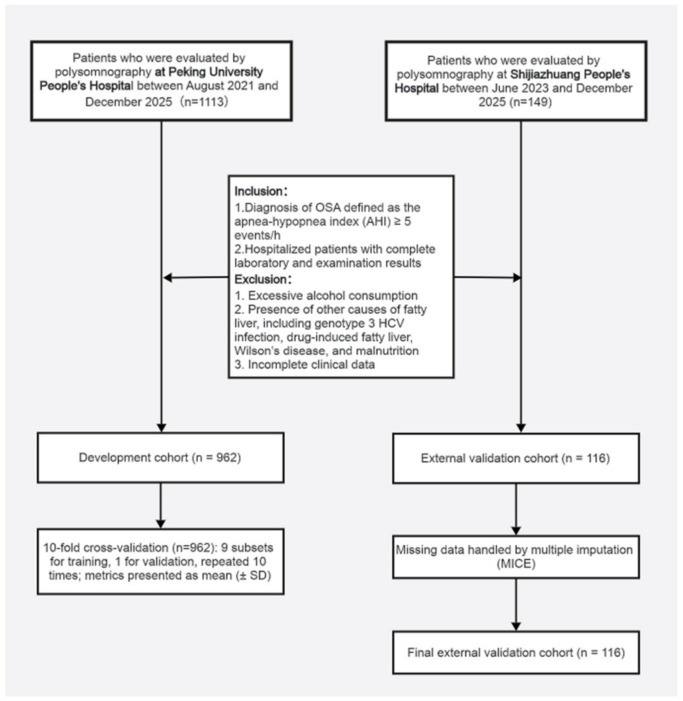
Flowchart of the procedure.

Patients with OSA from the Department of Geriatrics, Peking University People’s Hospital served as the development set. After excluding four participants without abdominal ultrasound data, a total of 962 participants were included. Patients with OSA from the Department of Geriatrics, Shijiazhuang People’s Hospital served as the external validation set, with a final inclusion of 116 participants after data cleaning (Figure 1).

Statistical analyses were performed using R Studio (version 4.5.1). Normally distributed continuous variables were presented as mean ± standard deviation and compared using independent samples *t*-test or analysis of variance. Non-normally distributed continuous variables were presented as median (Q1, Q3) and compared using the Mann–Whitney U test or Wilcoxon test. Categorical variables were presented as frequencies or percentages and compared using the chi-square test or Fisher’s exact test. With MAFLD as the binary outcome variable, sample size was assessed based on the principles of Riley et al. (2020) [30] for prediction model development, ensuring adequate event numbers and expected model performance (EPV target ≥ 10, preferably ≥ 20). The initial screening of 43 candidate predictors with 519 events (EPV ≈ 12) fell below the recommended threshold for stable multivariable model development. While LASSO regression and bootstrap stability testing were employed to mitigate overfitting risk, these techniques reduce but do not eliminate the possibility of chance findings. The simplified two-variable model was therefore prioritized to ensure adequate EPV and model stability.

Missing data were minimal across candidate variables (all <5% except for four patients missing the outcome variable, who were excluded). Multiple imputation by chained equations (MICE) was performed with m = 5 imputations and 20 iterations per dataset. All candidate variables were included in the imputation model to preserve relationships between variables. The imputation was conducted under the assumption of missing at random (MAR), which is reasonable given that missingness was primarily due to routine clinical testing patterns rather than disease severity. Formal missing data mechanism testing (e.g., Little’s MCAR test), complete case sensitivity analyses, and formal imputation diagnostics (e.g., kernel density plots comparing imputed and observed distributions) were not performed due to the retrospective nature of data extraction and technical limitations of the electronic medical record system. This is acknowledged as a limitation.

Candidate variables were first screened using univariate logistic regression, with assessment of multicollinearity (variance inflation factor, VIF). LASSO regression (10-fold cross-validation) was subsequently applied for variable dimensionality reduction, combined with bootstrap stability testing to select variables for inclusion in multivariate logistic regression. A fixed random seed (2026) was used throughout. The final prediction model was constructed. Model discrimination was assessed using the area under the receiver operating characteristic curve with 95% confidence intervals. Calibration was evaluated using calibration curves, calibration-in-the-large, calibration slope, and the Brier score, and the Hosmer–Lemeshow test was reported. Internal validation was performed using 10-fold cross-validation and bootstrap resampling (1000 iterations) with optimism correction. External validation was conducted in an independent cohort to evaluate AUC and calibration. The original (unrecalibrated) and recalibrated performances were both reported transparently.

Model comparisons were performed using the DeLong test for AUC comparisons, along with comparisons of calibration performance. Clinical utility was assessed using decision curve analysis (DCA), clinical impact curves (CICs), and net benefit. Within the clinically relevant threshold probability range of 0.30–0.70, the optimal threshold was determined based on the Youden index combined with clinical plausibility, and risk stratification (low/moderate/high) was performed accordingly. Sensitivity analyses included robustness testing across different thresholds and subgroup analyses stratified by sex, age, BMI, and OSA severity. A two-sided *p*-value < 0.05 was considered statistically significant.

The analytical workflow was conducted using the following R packages and parameters: glmnet (version 4.1-7) for LASSO regression with 10-fold cross-validation; rms (version 6.5-0) for model validation and calibration; pROC (version 1.18.0) for ROC analysis and DeLong test; rmda (version 1.6) for decision curve analysis. The random seed was fixed at 2026 throughout all resampling procedures. These details are provided to enable independent replication by researchers with access to similar datasets.

## 3. Results

Prior to model development, 43 candidate predictors were pre-selected based on clinical relevance and previous studies. Sample size for the binary prediction model was assessed using the Riley method. With 45 candidate parameters and an outcome event rate of 53.94%, the required minimum sample sizes were 7873, 3821, and 2469 when Cox-Snell R^2^ was set to 0.05, 0.10, and 0.15, respectively, all exceeding the actual effective sample size of 962 in this study [30]. Therefore, the sample size was insufficient to include all candidate variables simultaneously in the final model, necessitating variable selection through univariate analysis, LASSO regression, and multivariate regression. The simplified two-variable model was prioritized to ensure adequate events-per-variable ratio (EPV) and model stability.

A total of 966 OSA patients were initially included in the development set. After data quality review, four patients were excluded due to missing outcome variable (MAFLD), leaving 962 patients for model development and validation. For the external validation set, missing data were handled using predictive mean matching under a multiple imputation framework, generating five complete imputed datasets (m = 5). The chain equations were iterated 20 times per dataset to ensure algorithm convergence. Imputation quality was assessed by comparing the distribution of imputed values with observed values to ensure preservation of the original data distribution and avoid systematic bias.

### 3.1. Baseline Characteristics

Compared with the development set, the external validation set had a higher proportion of males (79% vs. 57%, *p* < 0.001, SMD = 0.49), younger age (median 54.5 vs. 67.0 years, *p* < 0.001, SMD = 0.83), and greater height, weight, and BMI (*p* < 0.001; SMD for height and weight: 0.51 and 0.61, respectively; SMD for BMI: 0.42). OSA severity distribution differed significantly between the two cohorts (*p* < 0.001, SMD = 0.74), while OAI and mean SpO_2_ showed no significant differences (*p* = 0.208 and 0.384, respectively). Regarding laboratory parameters, the external validation set had higher levels of LDL-C, FPG, TC, WBC, Hb, PLT, CRP, and INR, and lower eGFR (most *p* < 0.05, maximum SMD ~0.82), while differences in AST, Alb, TBIL, Cr, LY, MPV, and TG were not significant (*p* > 0.05). Overall, several SMD values exceeded 0.2 (Table 1), indicating baseline imbalance between the two cohorts, consistent with real-world demographic variations across independent centers. All MAFLD diagnoses were confirmed by abdominal ultrasonography. No patients were diagnosed by liver biopsy alone.

### 3.2. Stepwise Screening of Candidate Variables and Model Development

Univariate analysis showed that TyG index (OR ≈ 2.9, widest 95% CI) and TG (OR ≈ 1.9) exhibited the strongest positive associations, followed by ALT (OR ≈ 1.9) and TG/HDL-C (OR ≈ 1.8), while age (OR close to 1.0) and lowest SpO_2_ (OR < 1.0) had smaller effect sizes. Twenty-six variables, including TyG index, TG, OSA severity, lymphocyte count, diabetes, antihypertensive medication, hypertension, alcohol consumption, BMI, WBC, CAI, ALT, Hb, REI, ODI, hypoglycemic medication, eGFR, LDL-C, CRP, PLT, mean SpO_2_, longest apnea time, PLR, MPV, longest hypopnea time, lowest SpO_2_, and sex, reached statistical significance (*p* < 0.05) and proceeded to collinearity diagnostics. After variance inflation factor (VIF) assessment, three variables with high collinearity (hypertension, VIF = 35.71; DM, VIF = 16.3; TG, VIF = 14.15) were excluded, leaving 23 variables, all with VIF < 10.

**Figure 2:**
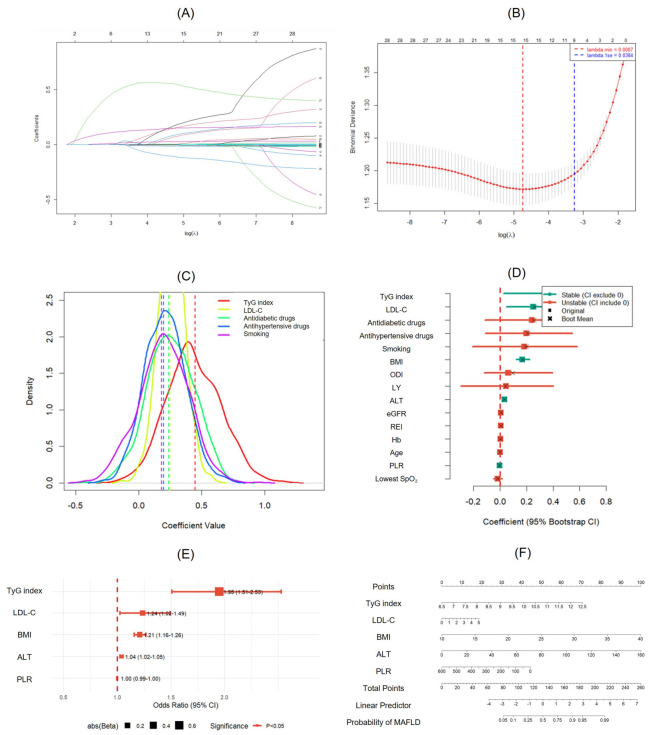
(**A**) Distribution of LASSO coefficients for each candidate variable. Each curve represents a different variable. The left ordinate denotes the coefficient values, and a coefficient of 0 indicates that the corresponding variable was not included in the model. LASSO, Least Absolute Shrinkage and Selection Operator. (**B**) Plot of Log (λ) (Logarithm of the Tuning Parameter) versus partial likelihood deviance based on LASSO Cross-Validation. At the top of the plot is the number of key feature variables corresponding to different Log (λ) values. The left dashed line represents the Log (λ) value corresponding to the minimum deviance, and the right dashed line denotes the Log (λ) value within one standard error of the minimum deviance. (**C**) Bootstrap coefficient distributions. Vertical dashed lines indicate estimates from the original sample. (**D**) Bootstrap stability assessment. Green indicates stable variables and red indicates unstable variables. (**E**) Multivariable logistic regression forest plot (**F**) Nomogram for risk prediction of MAFLD in OSAHS.

LASSO regression was used for variable selection, with the optimal penalty parameter λ selected via 10-fold cross-validation based on minimizing partial likelihood deviance or mean squared error. The lambda.min model included 15 non-zero coefficient variables, with TyG index having the highest coefficient, suggesting insulin resistance as the strongest diagnostic indicator of MAFLD. When λ was optimized, some coefficients were compressed to zero, enabling variable selection. Lambda.1se, located at −log(λ) ≈ 3.26, corresponded to a more parsimonious model with nine non-zero coefficient variables (within one standard error of the minimum deviance). At −log(λ) ≈ 4.75 (λ = 0.0087), the model achieved the minimum cross-validation error (binomial deviance ≈ 1.17), retaining 15 non-zero coefficient variables (Figure 2A,B).

To assess variable stability, bootstrap resampling (R = 1000, fixed random seed 2026) was performed on the 15 LASSO-selected variables. Only five variables demonstrated stable effects: TyG index (β = 0.450, 95% CI: 0.026–0.859), LDL-C (β = 0.249, 95% CI: 0.050–0.469), BMI (β = 0.167, 95% CI: 0.121–0.221), ALT (β = 0.032, 95% CI: 0.018–0.050), and PLR (β = −0.004, 95% CI: −0.008 to −0.001). All categorical variables (antihypertensive medication, hypoglycemic medication, smoking, etc.) were excluded, suggesting that after adjusting for metabolic indicators, the independent diagnostic value of these factors was limited. PLR was the only variable with a negative association, indicating a protective factor for MAFLD, consistent with previous studies, while the remaining indicators were risk factors [21] (Figure 2C,D).

A multivariate logistic regression model was constructed based on the five bootstrap-stable variables. The model equation was: Logit(P) = −11.206 + 0.668 × TyG index + 0.211 × LDL-C + 0.189 × BMI + 0.036 × ALT − 0.004 × PLR. The TyG index demonstrated the strongest independent association effect (OR = 1.95, 95% CI: 1.51–2.53, *p* < 0.001), followed by LDL-C (OR = 1.24, 95% CI: 1.02–1.49, *p* = 0.028) and BMI (OR = 1.21, 95% CI: 1.16–1.26, *p* < 0.001), consistent with previous studies [8]. Each 1 U/L increase in ALT was associated with a 3.6% increase in odds (OR = 1.04, 95% CI: 1.02–1.05, *p* < 0.001). PLR was the sole protective factor (OR = 0.996, 95% CI: 0.993–0.999, *p* = 0.004), suggesting that a higher platelet-to-lymphocyte ratio may reduce MAFLD risk (Figure 2E). The overall model performance was satisfactory (Nagelkerke R^2^ = 0.279, AIC = 1114.01). The likelihood ratio test indicated that the combined estimation of the five variables was significantly superior to the null model (χ^2^ = 225.59, df = 5, *p* < 0.001).

The full five-variable model (TyG + LDL-C + BMI + ALT + PLR) achieved an AUC of 0.770 (95% CI: 0.742–0.798) in the development set and 0.798 (95% CI: 0.718–0.878) in external validation, with a Brier score of 0.212 and calibration slope of 0.987. The simplified TyG-ALT model achieved an AUC of 0.714 (95% CI: 0.685–0.744) in the development set and 0.783 (95% CI: 0.701–0.866) in external validation, with a Brier score of 0.215 and calibration slope of 1.000. The discriminative difference was minimal (ΔAUC = 0.056 in development, 0.015 in external validation; DeLong test *p* = 0.084). The simplified model demonstrated superior calibration (slope 1.000 vs. 0.987) and a substantially higher events-per-variable ratio (259.5 vs. 103.8). Decision curve analysis showed comparable net benefit within the clinically relevant threshold range of 0.30–0.70. Net reclassification improvement was not calculated as the outcome is binary and the models are nested.

Although the full five-variable model demonstrated marginally higher discrimination, the simplified TyG-ALT model was selected for the following reasons: (a) the discriminative loss was minimal and non-significant; (b) the simplified model achieved superior calibration (slope closer to 1.0); (c) the simplified model avoids overfitting with a substantially higher events-per-variable ratio (259.5 vs. 103.8); (d) TyG and ALT are routinely available in primary care without additional cost, whereas LDL-C and PLR require fasting samples and specialized assays; (e) the simplified model is more clinically interpretable and actionable at the point of care.

A nomogram was constructed based on the model coefficients to facilitate individualized clinical prediction (Figure 2F). The final simplified model equation is:

Logit(P) = −8.742 + 0.624 × TyG index + 0.038 × ALT where TyG index = ln[TG (mg/dL) × FPG (mg/dL)/2]

Application method: For each variable in an OSA patient, a vertical line is drawn to determine the corresponding score. The scores for all predictors are summed, and the corresponding estimated probability of prevalent MAFLD is identified. For example, TyG = 10.2 (45 points) + LDL-C = 3.4 (12 points) + BMI = 28 (60 points) + ALT = 50 (30 points) + PLR = 120 (−1 point) yields a total score of approximately 150 points, corresponding to a predicted probability of approximately 75%.

### 3.3. Internal Validation

**Figure 3:**
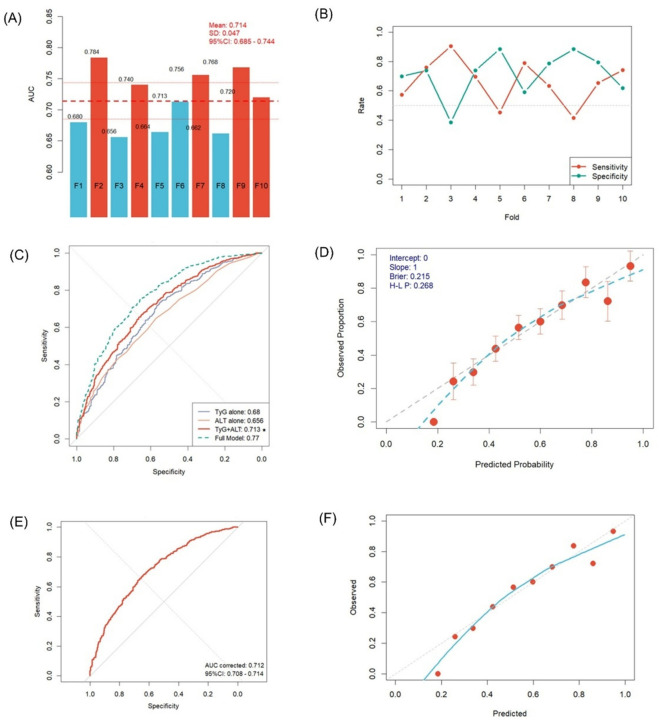
(**A**) Distribution of AUC values across cross-validation folds. Bars are color-coded by performance relative to the mean AUC (0.714): red indicates above-average performance, blue indicates below-average. Folds 2 and 9 demonstrated optimal performance, whereas folds 3 and 8 showed relatively poor discrimination. (**B**) Sensitivity–specificity trade-off by fold. Fold 3 exhibited high sensitivity but low specificity, while fold 8 showed the opposite pattern (low sensitivity, high specificity). In contrast, folds 2 and 9 achieved an optimal balance between sensitivity and specificity. (**C**) Predictive performance of different models for MAFLD in OSA patients: ROC curves in the development cohort. The AUC of the Full Model was 0.771, and the AUC of TyG+ALT was 0.713 *. (**D**) Calibration curve of the TyG-ALT model. (**E**) Optimism-corrected ROC curve after bootstrap validation (R = 1000). (**F**) Optimism-corrected calibration curve (Bootstrap, R = 1000).

A simplified diagnostic model based on TyG index and ALT was constructed. In the development set (*n* = 962), the AUC was 0.713 (95% CI: 0.681–0.745). Compared with TyG alone (AUC = 0.680, *p* = 0.0005) and ALT alone (AUC = 0.656, *p* = 0.0002), the combined model significantly improved diagnostic performance and achieved 92.6% of the discriminative ability of the full model (AUC = 0.770) (Figure 3C).

Internal validity of the TyG + ALT model was assessed using 10-fold cross-validation and bootstrap optimism correction. In 10-fold cross-validation, the development set (*n* = 962) was randomly partitioned into 10 subsets. In each iteration, nine subsets were used for training and one for validation, repeated 10 times to ensure that each sample was validated once. Final performance metrics were reported as the mean (±standard deviation) across the 10 folds. This approach more fully utilizes the data and reduces sampling variability compared with a single 7:3 split. Results showed that the maximum SMD across folds ranged from 0.175 to 0.341, the mean SMD ranged from 0.0648 to 0.0945, and the median SMD ranged from 0.0542 to 0.0829. The proportion of variables with SMD > 0.10 ranged from 19.6% to 37.5%, while the proportion with SMD > 0.20 was low (0–8.93%), indicating overall good balance across folds.

Internal validation yielded a mean AUC of 0.714 (SD = 0.047, 95% CI: 0.685–0.744), with AUC values across folds ranging from 0.656 to 0.784 and a coefficient of variation of 6.6%, suggesting moderate stability. Sensitivity ranged from 41.5% to 90.4%, and specificity ranged from 38.6% to 88.4%, reflecting variability in threshold trade-offs across different samples (Figure 3A,B). The model exhibited excellent calibration: calibration-in-the-large was 0, calibration slope was 1, Brier score was 0.215, and the Hosmer–Lemeshow test yielded *p* = 0.268, indicating good agreement between estimated probabilities and observed rates, with adequate fit. The optimal cutoff point was 0.524, corresponding to a sensitivity of 65.5% and specificity of 66.4% (Figure 3D).

Bootstrap optimism correction (R = 1000) showed an AUC of 0.712 (95% CI: 0.708–0.714), with an optimism of only 0.001. The difference between the two methods was 0.002, consistent with the 10-fold cross-validation results (difference < 0.01), confirming model stability (Figure 3E). The calibration curve showed that the fitted line was close to the 45° line, indicating good consistency between estimated probabilities and observed rates (Figure 3F).

### 3.4. External Validation

**Figure 4:**
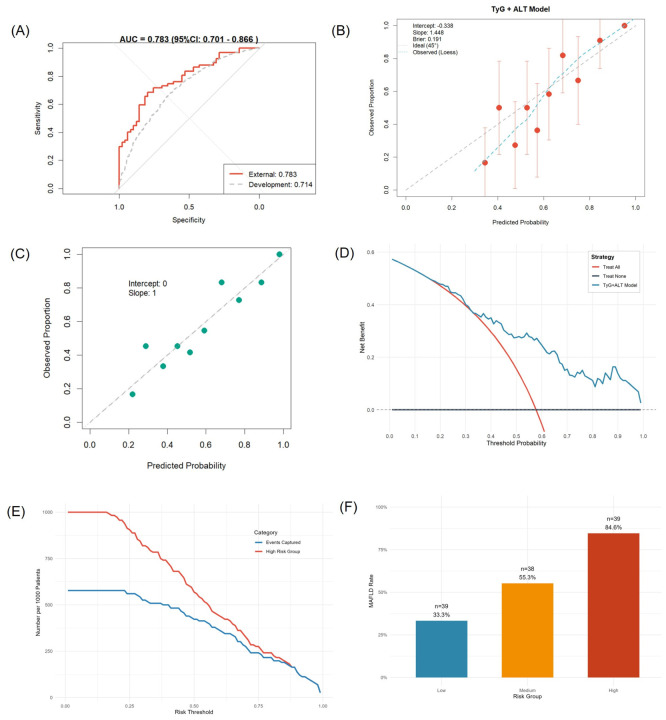
(**A**) ROC curve in the external validation cohort (AUC = 0.783, 95% CI: 0.701–0.866). (**B**) Calibration plot of the original model showing observed vs. predicted probabilities (intercept = −0.338, slope = 1.448, Brier score = 0.191). (**C**) Calibration plot after model recalibration (intercept = 0, slope = 1), demonstrating improved agreement between predicted and observed probabilities. (**D**) Decision curve analysis of the TyG-ALT model. (**E**) Clinical impact curve of the TyG-ALT model. (**F**) Observed MAFLD rates across risk stratification groups.

The TyG + ALT model demonstrated good discriminative ability in the external OSA validation cohort (*n* = 116), with an AUC of 0.783 (95% CI: 0.701–0.866), which was superior to its performance in the development set (Figure 4A). The wide confidence intervals reflect statistical uncertainty due to the small sample size. However, the model exhibited systematic calibration drift (intercept −0.338, slope 1.448, Brier score 0.191), indicating overestimation of MAFLD risk (Figure 4B). This bias was eliminated after simple linear logistic recalibration, which improved the intercept to 0.000, the slope to 1.000, and reduced the Brier score to 0.187, achieving ideal calibration (Figure 4C). The original and recalibrated performance are presented transparently. The recalibrated model can be used for MAFLD risk stratification in this population, but the unrecalibrated model should not be universally applied across centers without local validation. The recalibrated model can be used for MAFLD risk stratification in this population. Based on clinical utility considerations (sensitivity > 70% and specificity > 60%), the recommended threshold was 0.482. The optimal probability threshold based on the Youden index was 0.564, yielding a sensitivity of 68.7% and specificity of 79.6%.

### 3.5. Clinical Utility

Within the clinically relevant threshold probability range of 0.30–0.70,decision curve analysis showed that in the external validation set, the TyG index combined with ALT diagnostic model was superior to both treat-all and treat-none strategies across a threshold probability range of 0.17–0.99 (Figure 4D). At the optimal threshold of 0.564 (Youden index), the model identified high-risk patients for targeted ultrasound referral while avoiding unnecessary testing in low-risk patients. This threshold balances the consequences of false positives (unnecessary ultrasound and patient anxiety) against false negatives (missed MAFLD cases). Compared with current screening based on BMI ≥ 24 kg/m^2^ or elevated ALT alone, the TyG-ALT model provides continuous risk estimation and allows threshold customization based on local resource availability. However, whether this approach improves outcomes or cost-effectiveness compared with standard care requires prospective evaluation.

The clinical impact curve indicated that at a threshold of 0.564, approximately 400 out of 1000 patients were identified as high-risk, capturing about 68% of true event cases (approximately 270 out of 1000) while avoiding unnecessary intervention in 600 low-risk patients (Figure 4E). Patients in the external validation set were stratified into low, moderate, and high-risk groups based on tertiles of the recalibrated estimated probability. The MAFLD incidence rates were 33.3% in the low-risk group (*n* = 39, probability range 16.9–44.9%), 55.3% in the moderate-risk group (*n* = 38, probability range 45.2–69.7%), and 84.6% in the high-risk group (*n* = 39, probability range 69.9–100%), demonstrating a clear dose–response relationship. The event rate in the high-risk group was 2.54 times that of the low-risk group (84.6% vs. 33.3%), indicating good risk stratification capability of the model (Figure 4F).

### 3.6. Sensitivity Analysis

**Figure 5:**
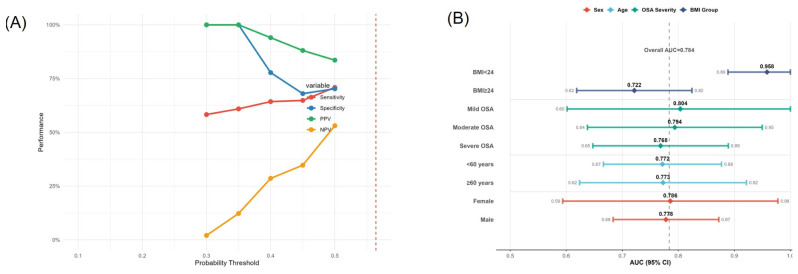
(**A**) Sensitivity analysis: Performance metrics across different probability thresholds. (**B**) Subgroup analysis of TyG + ALT model in external validation cohort.

To assess the robustness of the findings to parameter or methodological choices, and to evaluate uncertainty and potential bias, robustness testing across different thresholds and subgroup analyses stratified by sex, age, BMI, and OSA severity was conducted. The results showed that across the clinically relevant threshold range of 0.30–0.50, sensitivity, specificity, positive estimated value, and negative estimated value exhibited stable trends with changes in cutoff values, indicating that model performance was insensitive to cutoff selection. Even near the optimal Youden index cutoff (0.564, indicated by the red dashed line), performance did not fluctuate dramatically (Figure 5A).

Subgroup analyses revealed no bias by sex or age: AUC values for males and females were 0.778 and 0.786, respectively (difference < 0.01); for age < 60 years and ≥60 years, AUC values were 0.772 and 0.773, respectively (difference 0.001). All confidence intervals overlapped with the overall AUC (0.784), suggesting that the model maintains stable discriminative ability across different sex and age groups, with no significant subgroup effects. Furthermore, AUC values for mild, moderate, and severe OSA were 0.804, 0.794, and 0.768, respectively, all within an acceptable range. Although the AUC was slightly higher in mild OSA, the confidence intervals overlapped, and no trend of decreasing performance with increasing disease severity was observed (Figure 5B).

**Figure 6:**
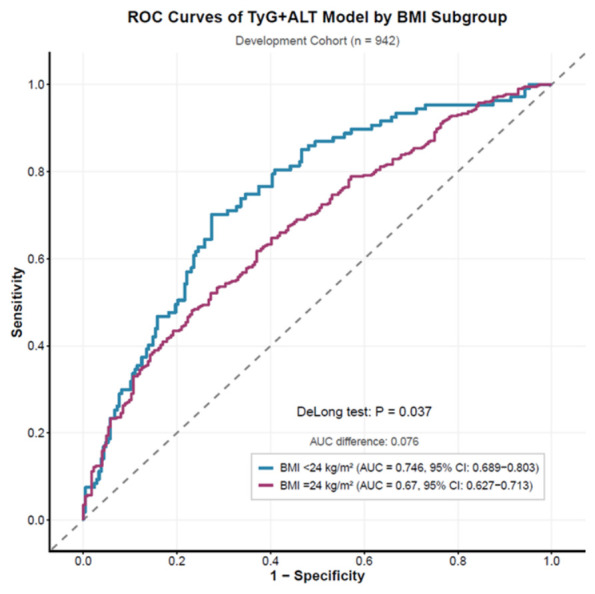
ROC curves stratified by BMI categories in the development cohort.

However, significant heterogeneity was observed in BMI stratification. In the BMI < 24 kg/m^2^ subgroup (*n* = 289, 89 MAFLD cases, 30.8% prevalence), the AUC was 0.958 (95% CI: 0.889–1.000). In the BMI ≥ 24 kg/m^2^ subgroup (*n* = 673, 430 MAFLD cases, 63.9% prevalence), the AUC was 0.722 (95% CI: 0.619–0.824). The exceptionally high AUC in the BMI < 24 kg/m^2^ subgroup (0.958) should be interpreted with substantial caution. This finding may reflect: (a) small sample size (*n* = 289) and limited events (*n* = 89), resulting in wide confidence intervals and unstable estimates; (b) spectrum bias, as lean MAFLD cases may represent a more homogeneous, metabolically distinct phenotype with clearer metabolic dysfunction signals; (c) potential overfitting despite cross-validation. Calibration could not be reliably assessed in the BMI < 24 subgroup due to a small sample size. The DeLong test indicated a statistically significant AUC difference (*p* = 0.037), but the interaction test showed no significant interaction between BMI and estimated probability (likelihood ratio test, *p* = 0.212), suggesting that the association between the TyG-ALT model and MAFLD remains consistent across BMI subgroups. This subgroup finding is exploratory and requires confirmation in larger cohorts specifically enriched for lean MAFLD before any clinical claims can be made (Figure 6).

However, significant heterogeneity was observed in BMI stratification. The BMI < 24 kg/m^2^ subgroup exhibited excellent performance, with an AUC of 0.958 (95% CI: 0.889–1.000), approaching perfect discrimination, suggesting that the model has a strong ability to identify MAFLD in low-BMI populations. In the BMI ≥ 24 kg/m^2^ subgroup, performance declined, with an AUC of 0.722 (95% CI: 0.619–0.824), though still above 0.70. To evaluate the modifying effect of BMI on model predictive performance, subgroup analysis was performed in the development set based on BMI < 24 kg/m^2^ and ≥24 kg/m^2^. The DeLong test indicated a statistically significant difference in AUC between the two subgroups (*p* = 0.037), but the interaction test showed no significant interaction between BMI and predicted probability (likelihood ratio test, *p* = 0.212), suggesting that the association between the TyG + ALT model and MAFLD remains consistent across BMI subgroups, though the model may be particularly prominent for screening lean MAFLD (Figure 6).

## 4. Discussion

This multicenter retrospective study cross-sectionally explored the association between the TyG-ALT composite index and MAFLD in patients with OSA. Previous studies have shown an association between NAFLD and OSA, with the incidence of NAFLD increasing with OSA severity [31]. However, the underlying mechanisms remain incompletely understood and may involve insulin resistance, oxidative stress, unfolded protein response, lipid metabolism disorders, and sleep deprivation resulting from chronic intermittent hypoxia. For example, studies have found that OSA patients and chronic intermittent hypoxia (CIH) model mice exhibit increased expression of proteins related to hepatic autophagy, endoplasmic reticulum stress, and lipogenesis [32,33,34,35]. Repetitive hypoxia/reoxygenation during apnea in OSA patients promotes reactive oxygen species production, leading to oxidative stress [36]. CIH promotes the degradation of Eepd1, a key DNA repair enzyme; liver-specific Eepd1 knockout mice exhibit exacerbated hepatic DNA damage, further aggravating liver inflammation, fibrosis, and progression of non-alcoholic steatohepatitis (NASH) [37,38,39]. Sleep disruption increases activity of the sympathetic nervous system, hypothalamic–pituitary–adrenal (HPA) axis, and oxidative stress responses, leading to elevated reactive oxygen species levels, promoting inflammation and pancreatic β-cell apoptosis, thereby contributing to glucose intolerance and insulin resistance [40].

This study constructed a composite diagnostic model based on ALT and TyG, establishing the significant diagnostic value and diagnostic significance of TyG-ALT. The model reveals the cascade mechanism by which OSA promotes MAFLD from the dual dimensions of liver injury and systemic metabolic disturbances, further suggesting the initiation of a vicious cycle of hypoxic stress–metabolic disorder–liver injury in OSA patients, serving as a marker for prevalent MAFLD. Using easily measurable indicators for disease case-finding facilitates primary care screening and risk control, providing a reference for clinical assessment.

Studies have shown that patients with NAFLD and normal BMI have similar risks of liver-related and non-liver-related events compared with overweight and obese NAFLD patients, both exhibiting metabolic dysfunction and risk of advanced fibrosis [41,42]. Moreover, patients with lean MAFLD have worse liver function outcomes and higher all-cause mortality risk [43]. Observational studies have indicated an association between OSA and MAFLD that is independent of obesity [44,45,46]. However, multiple studies have confirmed that obesity is a risk factor for OSA [47,48]; therefore, the role of BMI in the relationship between these two conditions cannot be overlooked. In exploratory subgroup analysis, we observed that in the BMI < 24 kg/m^2^ population, the model achieved an AUC of 0.958, but this finding requires confirmation in larger cohorts before clinical application.

Emerging evidence supports the utility of biomarker-based panels for MAFLD screening in high-risk populations, particularly when imaging resources are limited [29]. Our TyG-ALT model aligns with this paradigm by offering a simple, cost-effective alternative for initial risk stratification before confirmatory imaging.

Furthermore, MAFLD is a multisystem metabolic disease that not only leads to end-stage liver diseases such as decompensated cirrhosis and hepatocellular carcinoma but also increases the risk of type 2 diabetes, cardiovascular disease, chronic kidney disease, and non-liver malignancies [49,50,51,52]. Therefore, preventing MAFLD and its complications through non-pharmacological measures, and implementing intensified preventive strategies in high-risk populations—such as managing OSA and diabetes—are particularly important for the prevention and treatment of MAFLD and its comorbidities [53].

## 5. Strengths and Limitations

This study employed rigorous methodological approaches, including univariate logistic regression, multicollinearity diagnostics, LASSO regression with 10-fold cross-validation, and bootstrap stability testing to select variables for multivariate logistic regression. Five predictors were ultimately selected: TyG index, LDL-C, BMI, ALT, and PLR. While the diagnostic performance of TyG index combined with BMI has been validated in previous studies, ALT directly reflects liver inflammation and injury, whereas LDL-C, as a lipid metabolism indicator, has limitations in reflecting liver injury driven by intermittent hypoxia in OSA patients. Currently, there is a lack of diagnostic models combining TyG and ALT to assess the risk of metabolic liver disease in the OSA population. This study fills this gap by performing rigorous internal validation using 10-fold cross-validation and bootstrap optimism correction, and demonstrating good discriminative ability in an independent external validation cohort. Although initial external calibration showed deviation, it was significantly improved by recalibration, indicating that the model requires local updating before implementation in new clinical settings.

The limitations of this study are as follows: First, the study design is retrospective and cross-sectional. The model estimates the probability of prevalent MAFLD at the time of clinical assessment rather than predicting future incident MAFLD. Therefore, the terms “prediction” and “predictive model” refer to statistical estimation of current disease status, not longitudinal forecasting. Causal inferences and assessment of intervention effects are not supported by this design. Second, the reference standard (abdominal ultrasonography) has moderate sensitivity for mild steatosis and cannot assess steatohepatitis or fibrosis. MRI-PDFF and CAP were unavailable. Formal interobserver reliability assessment was not performed. These limitations may result in misclassification of mild MAFLD cases and underestimation of disease severity. Third, the initial screening of 43 candidate predictors with 519 events (EPV ≈ 12) fell below the recommended threshold for stable multivariable model development. While LASSO regression and bootstrap stability testing were employed to mitigate overfitting risk, these techniques reduce but do not eliminate the possibility of chance findings. The simplified two-variable model was therefore prioritized to ensure adequate EPV (259.5) and model stability. Future studies with larger cohorts (target *n* > 2500) are needed to validate the full five-variable model. Fourth, the external validation cohort was small (*n* = 116, 67 MAFLD cases), resulting in wide confidence intervals for performance metrics. The substantial baseline imbalance between cohorts (SMD > 0.2 for age, sex, BMI, OSA severity, and multiple laboratory parameters) limits the generalizability of performance estimates. These findings should be interpreted as a preliminary stress test of model transportability rather than definitive evidence of broad applicability. Fifth, model recalibration was necessary in the external cohort (intercept = −0.338, slope = 1.448 before recalibration), indicating that the unrecalibrated model overestimates MAFLD risk in populations with different baseline characteristics. The unrecalibrated model should not be directly deployed across centers without local validation and updating. Sixth, the subgroup finding in BMI < 24 kg/m^2^ (AUC = 0.958) is exploratory and potentially unstable due to small sample size (*n* = 289, 89 events). Calibration could not be reliably assessed in this subgroup. This finding requires confirmation in larger cohorts specifically enriched for lean MAFLD before any clinical claims can be made. Seventh, although various confounders were adjusted for, residual confounding factors may still exist. Future prospective, multicenter, nationwide cohort studies are needed to more comprehensively evaluate the role of TyG-ALT and other indicators in MAFLD development in OSA patients.

## 6. Conclusions

The TyG index combined with ALT is independently associated with prevalent MAFLD in patients with OSA. The simple diagnostic risk-stratification model based on these two indicators demonstrates good discriminative ability, calibration, and clinical utility, serving as an effective tool for case-finding and risk stratification of prevalent MAFLD in the OSA population, facilitating timely identification of high-risk individuals for confirmatory imaging and personalized management.

## Figures and Tables

**Table 1:** Distribution of base line characteristics in the development and external validation groups [(%)/M (P_25_, P_75_)].

Independent Variable	Level	Development Set (N = 962)	External Validation Set (N = 116)	95% CI	SMD	*p* Value
Gender				0.29, 0.68	0.49	<0.001
	Man	552 (57)	92 (79)			
	Woman	410 (43)	24 (21)			
Age (years)		67.00 (59.00, 74.00)	54.50 (44.50, 66.00)	0.63, 1.00	0.83	<0.001
Height (m)		1.67 (1.60, 1.72)	1.72 (1.65, 1.76)	−0.70, −0.32	−0.51	<0.001
Weight (kg)		70.00 (62.00, 79.25)	79.00 (70.00, 88.00)	−0.80, −0.41	−0.61	<0.001
OSA severity				0.55, 0.94	0.74	<0.001
	Mild	440 (46)	22 (19)			
	Moderate	324 (34)	36 (31)			
	Severe	195 (20)	58 (50)			
REI		16.30 (10.20, 26.80)	30.05 (17.30, 45.85)	−0.98, −0.59	−0.79	<0.001
OAI		4.10 (2.00, 8.20)	4.60 (1.20, 15.70)	−0.72, −0.33	−0.53	0.208
CAI		0.90 (0.40, 1.80)	0.00 (0.00, 0.15)	0.53, 0.92	0.73	<0.001
Lowest SpO_2_ (%)		84.00 (80.00, 87.00)	82.50 (77.00, 86.00)	0.14, 0.52	0.33	0.021
Longest apnea time (s)		59.50 (50.50, 71.50)	40.40 (30.10, 54.60)	0.08, 0.47	0.28	<0.001
Longest hypopnea time (s)		58.00 (54.50, 59.50)	65.95 (50.65, 84.60)	−0.94, −0.54	−0.74	<0.001
Mean SpO_2_ (%)		94.00 (92.00, 95.00)	94.00 (92.00, 94.50)	−0.11, 0.27	0.08	0.384
ODI4 (events/h)		12.90 (7.50, 22.90)	20.80 (11.80, 38.95)	−0.79, −0.40	−0.60	<0.001
ALT (U/L)		17.00 (13.00, 25.00)	25.00 (18.50, 36.00)	−0.75, −0.36	−0.56	<0.001
AST (U/L)		19.00 (16.00, 24.00)	20.00 (17.00, 24.00)	−0.39, 0.00	−0.19	0.092
GGT (U/L)		22.50 (16.00, 35.00)	36.00 (20.50, 50.00)	−0.48, −0.10	−0.29	<0.001
Albumin (g/L)		41.70 (39.70, 43.90)	42.10 (39.10, 43.80)	−0.14, 0.24	0.05	0.783
Total bilirubin (μmol/L)		13.40 (10.85, 16.55)	13.70 (11.05, 18.45)	−0.33, 0.05	−0.14	0.176
Creatinine (μmol/L)		68.00 (58.00, 80.50)	68.00 (60.00, 76.00)	−0.20, 0.19	0	0.526
eGFR (mL/min/1.73 m^2^)		91.01 (79.06, 98.51)	101.33 (93.03, 109.40)	−1.00, −0.62	−0.82	<0.001
Uric acid (μmol/L)		350.00 (293.00, 410.00)	371.00 (315.50, 428.00)	−0.34, 0.05	−0.14	0.056
FPG (mmol/L)		5.11 (4.64, 5.95)	5.30 (4.90, 5.90)	−0.14, 0.25	0.05	0.031
TC (mmol/L)		4.20 (3.52, 4.96)	4.43 (3.76, 5.22)	−0.33, 0.06	−0.13	0.039
TG (mmol/L)		1.36 (0.97, 1.96)	1.52 (0.96, 2.13)	−0.24, 0.14	−0.05	0.329
HDL-C (mmol/L)		1.14 (0.97, 1.35)	1.11 (0.93, 1.27)	−0.12, 0.26	0.07	0.039
LDL-C (mmol/L)		2.43 (1.86, 3.07)	2.96 (2.37, 3.45)	−0.70, −0.31	−0.50	<0.001
WBC (×10^9^/L)		5.50 (4.60, 6.50)	6.00 (4.88, 7.13)	−0.52, −0.13	−0.33	<0.001
Lymphocyte (×10^9^/L)		1.70 (1.40, 2.10)	1.74 (1.39, 2.07)	−0.19, 0.19	0	0.53
Hemoglobin (g/L)		134.00 (124.00, 144.00)	144.90 (134.25, 152.95)	−0.78, −0.39	−0.59	<0.001
Platelet (×10^9^/L)		194.00 (163.00, 227.00)	233.50 (185.50, 263.00)	−0.73, −0.34	−0.54	<0.001
MPV (fL)		8.50 (7.85, 9.30)	8.40 (7.84, 9.06)	−0.26, 0.13	−0.06	0.269
CRP (mg/L)		0.85 (0.50, 2.00)	1.28 (0.64, 3.40)	−0.27, 0.12	−0.08	0.002
INR		0.96 (0.93, 1.00)	0.99 (0.95, 1.04)	−0.16, 0.23	0.03	<0.001
MAFLD				−0.12, 0.27	0.08	0.596
	No	443 (46)	49 (42)			
	Yes	519 (54)	67 (58)			
Hypertension				0.09, 0.48	0.28	0.008
	No	338 (35)	26 (22)			
	Yes	624 (65)	90 (78)			
Diabetes mellitus				−0.01, 0.37	0.18	0.094
	No	608 (63)	83 (72)			
	Yes	354 (37)	33 (28)			
Coronary heart disease				0.00, 0.38	0.19	0.055
	No	775 (80)	84 (72)			
	Yes	187 (20)	32 (28)			
Other diseases				0.57, 0.96	0.77	<0.001
	No	460 (48)	17 (15)			
	Yes	502 (52)	99 (85)			
Antihypertensive drugs				0.07, 0.46	0.26	0.009
	No	340 (35)	56 (48)			
	Yes	621 (65)	60 (52)			
Antidiabetic drugs				0.33, 0.72	0.53	<0.001
	No	617 (64)	100 (86)			
	Yes	344 (36)	16 (14)			
Antiplatelet drugs				0.13, 0.52	0.33	0.004
	No	705 (73)	100 (86)			
	Yes	256 (27)	16 (14)			
Long-term medication (other)				0.25, 0.63	0.44	<0.001
	No	529 (55)	39 (34)			
	Yes	432 (45)	77 (66)			
Smoking				0.00, 0.39	0.2	0.052
	No	709 (74)	75 (65)			
	Yes	253 (26)	41 (35)			
Alcohol consumption				−0.10, 0.29	0.09	0.389
	No	768 (80)	88 (76)			
	Yes	194 (20)	28 (24)			
BMI (kg/m^2^)		25.35 (23.05, 28.03)	27.09 (24.47, 29.73)	−0.62, −0.23	−0.42	<0.001
PLR		116.75 (91.70, 147.74)	128.07 (106.90, 158.71)	−0.46, −0.08	−0.27	<0.001
WBC/MPV		0.64 (0.54, 0.78)	0.73 (0.56, 0.89)	−0.24, 0.15	−0.04	<0.001
TG/HDL-C		1.21 (0.80, 1.83)	1.34 (0.84, 2.21)	−0.28, 0.11	−0.09	0.109
TyG index		8.68 (8.26, 9.08)	8.75 (8.35, 9.13)	−0.28, 0.10	−0.09	0.322

## Data Availability

The clinical data supporting the findings of this study are derived from electronic medical records of Peking University People’s Hospital and Shijiazhuang People’s Hospital. Due to the privacy of the patients and the ethical restrictions, the raw data cannot be shared publicly. De-identified data may be available from the corresponding author, Jingtong Wang, upon reasonable request and subject to approval from the ethics committee.
